# Determinants of life satisfaction in adolescents with congenital or acquired heart disease: a nationwide cross-sectional study

**DOI:** 10.1186/s12889-024-20758-5

**Published:** 2024-11-28

**Authors:** Mohamad El-Chouli, Sidsel Marie Bernt Jørgensen, Daniel Mølager Christensen, Isabella Drachmann, Thomas Steen Gyldenstierne Sehested, Morten Winther Malmborg, Sandra Chamat-Hedemand, Caroline Sindet-Pedersen, Lars Idorn, Gunnar Hilmar Gislason, Thomas Alexander Gerds, Susan Ishøy Michelsen, Nina Føns Johnsen

**Affiliations:** 1 Danish Heart Foundation, Vognmagergade 7, Copenhagen, 1120 Denmark; 2 Department of Cardiology, Roskilde University Hospital, Zealand, Denmark; 3 Department of Public Health & Center for Healthy Aging, University of Copenhagen, Copenhagen, Denmark; 4 Department of Cardiology, Copenhagen University Hospital, Herlev and Gentofte, Hellerup, Denmark; 5 The Danish Knowledge Centre for Rehabilitation and Palliative Care, Amager and Hvidovre Hospital, Copenhagen, Denmark; 6 Department of Cardiology, Rigshospitalet, Copenhagen, Denmark; 7 Department of Pediatric Cardiology, Rigshospitalet, Copenhagen, Denmark; 8 Department of Clinical Medicine, University of Copenhagen, Copenhagen, Denmark; 9 Section of Biostatistics, University of Copenhagen, Copenhagen, Denmark; 10 National Institute of Public Health, University of Southern Denmark, Copenhagen, Denmark

**Keywords:** Congenital Heart Disease, Acquired Heart Disease, Adolescents, Determinants, Life satisfaction

## Abstract

**Background:**

We aimed to investigate how self-reported physical and cognitive limitations (challenges), self-efficacy, and social support interacted with life satisfaction in adolescents and young adults with congenital heart disease (CHD) or acquired heart disease, among whom life satisfaction may be impaired.

**Methods:**

“Adolescence with Heart Disease” was a cross-sectional, nationwide survey of patients with CHD or early acquired heart disease aged 15–25. Structural equation modeling was used to test the implied latent variable mediation model between the main outcome of interest (life satisfaction) and challenges, social support, and self-efficacy. The correlation factors between life satisfaction and the latent variables with 95% confidence intervals (CIs) were calculated.

**Results:**

A total of 1691 patients were included: 72% had CHD, 52% were females, the median age at response was 20 years [interquartile range: 18;23], and 69% reported high life satisfaction. In the univariate models, high life satisfaction was significantly associated with low challenges (-0.5 [CI: -0.6;-0.5]), high self-efficacy (0.8 [CI: 0.7; 0.8]), and high social support (0.4 [CI: 0.3; 0.5]). In the multivariate model, only self-efficacy remained significantly associated with life satisfaction (0.8 [CI: 0.7; 0.9]). Furthermore, there was a significant negative covariance between challenges and both self-efficacy (-0.67) and social support (-0.4), while the two latter variables had a positive covariance (0.55).

**Conclusions:**

In adolescents with heart disease, high life satisfaction was associated with high levels of self-efficacy. High levels of self-efficacy and social support attenuated the association between physical and cognitive challenges and life satisfaction. In this group that may face higher challenges than their peers, future interventions should aim to increase their self-efficacy to improve their life satisfaction, potentially through promoting social support.

**Supplementary Information:**

The online version contains supplementary material available at 10.1186/s12889-024-20758-5.

## Introduction

Many children with congenital heart disease (CHD) require multiple surgeries and close follow-up in the healthcare system, and many live with physical and neurocognitive limitations in their daily lives [1–5]. Treatment advances have led to a shift in disease management focus in this group from only including short-term cardiac care to long-term and complete care [6]. The transition from childhood to adulthood marks a particularly vulnerable period for these patients. Adolescents with heart disease must navigate somatic, psychosocial, and emotional changes while transitioning from the well-known pediatric care to adult care [7].

When investigating the well-being of patients, the chosen definition of quality of life determines the outcome. CHD patients have, especially as the lesions get more complex, more inhibited physical capabilities compared to healthy peers [8]. When comparing their quality of life from a physical function perspective, adult CHD patients showed a poorer quality of life compared to healthy controls [5]. A different approach, used in positive psychology, in defining the quality of life in patients with chronic diseases is looking at their overall life satisfaction, which refers to the patient’s subjective appraisal of their life [9–11]. Health-related quality of life has chronic patients, including those with CHD, at a disadvantage from baseline. Life satisfaction, however, compares them on a more level field compared to the background population due to focusing on strengths, rather than shortcomings [8]. A previous study found that while health-related quality of life was significantly associated with CHD severity, life satisfaction was not [12]. They also found that life satisfaction was associated with socio-demographic factors such as educational level and employment status. The relation between life satisfaction, physical and cognitive limitations, and promotive factors in adult patients with CHD has previously been investigated. [13] A study on adults with CHD in Belgium had a higher life satisfaction compared to healthy matched controls [9]. Meanwhile, another study based in Iran found the opposite [13]. Some of the determinants of worse life satisfaction in adult CHD patients in both studies were emotional distress, physical limitations, lower social support, and not having a job or career. Similar determinants were found in an international study by Apers et al. in adult CHD patients, which also found no country-specific differences [14]. However, unlike the physical limitations, the results on the association between cognitive limitations and life satisfaction have been inconsistent [15–18].

Unlike in adult CHD patients, literature on life satisfaction in adolescents with CHD is lacking, as many studies focus on health-related quality of life. One study found that quality of life among 74 adolescents and young adults with CHD was better compared to the general population [19]. Determinants of a better quality of life included higher physical function, fewer psychological limitations, and greater social support. Like the findings in adult CHD patients, no association was found between CHD severity and quality of life. Another study based in Iran on 80 adolescent and young adult CHD patients also reported that the complexity of the CHD lesion did not impact life satisfaction [20]. However, both studies had small sample sizes. Larger studies focusing on the determinants of life satisfaction in adolescents with CHD are therefore needed. Furthermore, acquired heart diseases such as arrhythmias and cardiomyopathies are less common than CHD in young individuals [21–24] and therefore not as prevalent in quality of life literature. Nevertheless, they can contribute significantly to morbidity and mortality in this group when they occur [21–24]. Investigating this group is, therefore, also important.

This study investigated whether physical and cognitive limitations, self-efficacy, and social support were associated with life satisfaction in Danish adolescents with congenital or acquired heart disease and, if so, how these factors interacted.

## Methods

### Study population

This study used data from a national, cross-sectional survey of Danish adolescents and young adults with congenital heart disease, the “Adolescence with Heart Disease” study [25]. Participants in the survey were identified using Danish nationwide administrative registries, which hold complete information on all contacts to Danish hospitals [26]. Data in the Danish nationwide registries were linked on an individual level by using the unique and permanent civil registration number, which is assigned to every Danish citizen at the date of birth or immigration. Patients with CHD or acquired heart disease were identified using primary and secondary diagnoses from in- and outpatient hospital contacts registered in Danish registries (Table S1). Comorbidities, including attention deficit hyperactivity disorder (ADHD), depression, anxiety, obsessive compulsive disorder (OCD), eating disorder, migraine, and asthma were identified in the survey response.

The inclusion criteria for the survey were 1) 15–25 years of age at the date of data retrieval in 2020 and 2) at least one contact with a Danish department of cardiology between January 1st, 2014, and December 31st, 2018, with a diagnosis of CHD or early acquired heart disease (including heart blocks, atrial or ventricular arrhythmia, cardiomyopathy, and pulmonary hypertension; the diagnosis codes are listed in Table S1). If a patient had both CHD and acquired heart disease, they were classified as CHD. Excluded were any diagnosis of intellectual disability disorder (Table S1). All included registries have been validated previously [27, 28]. A total of 3477 individuals were identified in the survey. Of these, 106 (3%) could not participate due to either cognitive difficulties or no access to the digital and secure government-issued mailbox. Of the remaining 3371, 49% answered the whole questionnaire, 11% answered parts of the questionnaire, and 40% did not answer. The response rate was 60%, corresponding to 2038 individuals.

Nonrespondents, compared with respondents, were significantly more likely to be male (61% vs 50%), descendants of immigrants (10% vs 5%), and to have primary school as the highest completed education level (69% vs 62%) [25].

### Questionnaire development and distribution

The questionnaire was developed in multiple steps. First, relevant topics to explore were identified via a review of the literature examining the daily lives of adolescents with CHD and their contact with education and healthcare systems. Second, qualitative interviews were conducted with individuals from the target group, parents of adolescents with CHD and experts from the healthcare system and the education system with contact with the target group. The aim of the interviews was to explore the implications of living with CHD in adolescents, both in regard to their daily life, lifestyle and contact with the education and healthcare system. Third, a preliminary questionnaire was developed based on the results of the literature review and the qualitative study. Since the main purpose of the survey was to describe and enable comparison of young people with and without heart disease, widely used items from large surveys among young people in Denmark were selected. The preliminary questionnaire was evaluated through the Delphi technique in e-mail consultations with a group consisting of the target group, their parents and experts working with the target group [29]. Third, the preliminary questionnaire was tested in cognitive interviews with the target group [30]. Finally, the preliminary questionnaire was pilot tested in a sample of 100 individuals from the study population. The final questionnaire encompassed questions regarding their daily lives, heart disease, and contact with the healthcare system. The questionnaire included both open- and closed-ended questions and internationally validated scales (i.e., self-rated health, New York Heart Association classification, and the transition scale, among others) [31–33]. The survey was sent to adult patients and the digital mailbox of parents to patients less than 18 years old, with electronic and telephone reminders sent to nonrespondents. To increase the response rate, an iPhone raffle among respondents was held, information material was sent out highlighting the importance of answering regardless of symptomology, and respondents could close the survey and continue later. The latter to facilitate the answering process for those with cognitive or physical limitations.

The data collection took place in January–February 2020 and August–September 2020.

### Outcome

Life satisfaction was measured by a previously used and validated modified version of the Cantril ladder, where the individual evaluates their overall life satisfaction [34, 35]. In the questionnaire, the participants answered the question, “*On this scale, 10 meaning the best life possible for you and 0 meaning the worst life possible for you, where do you currently feel you are?*”. The response options were 0–10, with a higher score corresponding to higher life satisfaction. High life satisfaction was defined as a score of 7 or above, while scores of 6 or below indicated low life satisfaction [34]. Due to its nonspecific nature, this measure of life satisfaction allows comparison across different responder groups [35].

### Potential determinants

Among adolescent CHD patients, physical limitations (such as physical pain or dyspnea during physical exertion), psychological limitations (such as the general ability to concentrate), and social support (i.e., from friends) have previously been associated with quality of life [19]. The same associations were found among adult CHD patients [9, 13, 14]. Previous studies reported a positive and strong association between life satisfaction and self-efficacy among healthy adolescents, which represents the individual’s belief in their own abilities to attain what they want [36–39]. Furthermore, higher self-efficacy appears to moderate the effect of social life-related stressors experienced by adolescents [37, 40]. In this study, we chose to look at physical and cognitive limitations, social support, and self-efficacy as determinants of life satisfaction.

The levels of physical and cognitive limitations were measured by three items used to construct a single latent variable, “challenges”. The first question concerned limitations in physical functioning and was measured by the New York Heart Association classification (Table S2) [31]: 1) “*Which of the following statements describe your ability to perform physical tasks*” with four response categories: none to severe physical limitations. Two questions measured the cognitive challenges: 1) “*Do you have difficulty concentrating in your daily life*” and 2) “*Do you find it difficult to remember what you need to do during the day?*” Both items were used in “*Health Behaviour in School-aged Children*”, unpublished. Response options ranged from “*never*” to “*often*” for both questions (Table S2).

*Self-efficacy*: Self-efficacy was measured using two questions related to self-confidence and belief in one’s own ability that were grouped as a single latent variable: 1) “*How much do you agree or disagree with this statement: I am good enough as I am*” with five corresponding to “Strongly disagree” and one corresponding to “Strongly agree” [41], and 2) “*How often can you follow through on what you set out to do?*” with a response scale of 1–5 with one corresponding to “Always” and five corresponding to “Never” (Table S2) [42–44]. The self-efficacy items used were developed at the National Institute of Public Health, Denmark. The face validity of the items was tested and considered high and the conceptual validity was checked by studying the correlation with several other measures of mental health (for example emotional symptoms and life satisfaction) [42]. Furthermore, the differential item function was low, suggesting a high construct validity [42]. The two items are used in the annual Danish National Well-being Questionnaire mandatory in school every year [45, 46]. This survey is developed by an expert group on behalf of the Danish Ministry of Children and Education and participation is mandatory for public schools and youth educations.

*Social support*: Social support was measured by the question: “*Who do you talk to when something is troubling you or you are upset?*” followed by multiple response options for parents/stepparents, siblings, friends, partner, personnel at school or workplace, and healthcare personnel (Table S2) [43]. This item is a revised version of an item from HBSC measuring confidentiality with parents [47].

### Statistical analyses

**Fig. 1:**
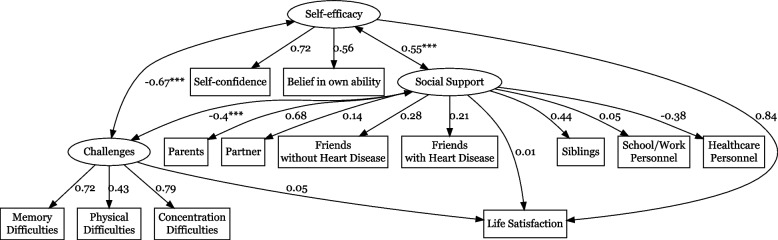
Depiction of the structural model of the relationships between self-reported physical and mental challenges, belief in one’s own ability and self-confidence, social support domains, and life satisfaction. Correlation factors on standardized scales are provided between variables. Asterisks (***) indicate significant covariance between latent variables

Challenges, self-efficacy, and social support are unobserved constructs (latent variables) hypothesized to underlie the item responses. Furthermore, each questionnaire item could have a different amount of response options (for instance, social-support related items had binary yes/no options, while concentration difficulties had a scale between 1–5) which would make direct correlations difficult. To analyze the associations between the latent variables and the outcome (life satisfaction), we used a structural equation model where the latent variables of social support, challenges, and self-efficacy were represented by the determinants listed in the previous section (Fig. 1). We used a standardized scale in the model to make different latent variables comparable across varying scales, achieving a unit-free interpretation of the regression coefficients. The structural equation model allows the incorporation of many different variables and analyzes the intercorrelation between them. Furthermore, it provides an estimate of the strength of the association, making it ideal for exploring associations in large datasets. We compared the different latent variables with varying scales using a standardized scale, where variables have a mean of 0 and a standard deviation of 1. The associations between the latent variables and outcome were evaluated using a unitless correlation factor, where a positive estimate indicates a positive association and a negative factor suggests a negative association. To describe the data used in the structural equation model, a heatmap showed life satisfaction against the sum of the determinants of the three latent variables.

The model was fitted using the diagonally weighted least squares estimation method, which provides robust standard errors and mean- and variance-adjusted test statistics [48]. We calculated the model goodness of fit indices: the scaled root mean square error of approximation (RMSEA) with 90% confidence intervals, the Tucker-Lewis Index (TLI), Standardized Root Mean Square Residual (SRMR), and the comparative fit index (CFI).

### Sensitivity analysis

To examine a potential effect modification, analyses were stratified by sex and age group (15–19 years, 20–25 years) at the time of diagnosis. An additional analysis with CHD patients classified according to severity was performed to examine whether the severity of CHD affected the associations.

We classified CHD patients into three mutually exclusive severity groups as defined by international guidelines, applying a hierarchical approach previously used [49–51]: 1) simple CHD [52], which refers to small lesions with an expected benign prognosis (defined as isolated atrial septal defect, isolated ventricular septal defect, isolated pulmonary stenosis, or isolated occluded ductus arteriosus), 2) moderate CHD (all CHDs not included in simple or severe CHD, such as atrioventricular septal defect, Ebstein’s anomaly, or more than one “simple” CHD), and 3) severe CHD [53], which refers to major malformations of the heart and great arteries that generally require intervention within the first year of life (defined as double-outlet ventricle, interrupted aortic arch, univentricular heart, pulmonary atresia, transposition of the great arteries, truncus arteriosus, presence of Eisenmenger syndrome, or other abnormalities of atrioventricular and ventriculoarterial connections such as ventricular inversion) (Table S1). Patients with unclassified CHD diagnosis (e.g., DQ248, “Other specified congenital malformations of the heart”) were not included in this analysis.

## Results

**Fig. 2:**
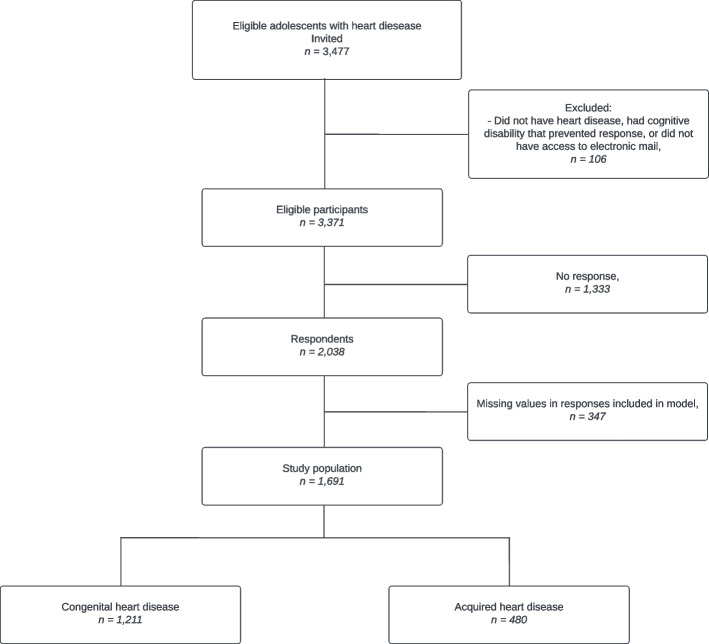
Flow chart depicting the inclusion, selection, and exclusion of the study population

Of the 2038 individuals participating in the “Adolescence with Heart Disease” survey, we excluded 347 (17%) for the present study due to missing values in at least one of the variables in our model, leaving a total of 1691 individuals for inclusion in the study (Fig. 2).

In the total population, 1211 (71.6%) of the respondents had CHD, while 480 (28.4%) had acquired heart disease (Table 1). Almost half of the population was male (48.0%), and the median age at the time of survey completion was 20 years. The most prevalent comorbidities were anxiety (14.4%) and migraine (14.2%), which were significantly higher in females than in males (Table 1).

**Table 1:** Baseline characteristics of the study population, stratified by sex

	**Males (n = 812)**	**Females** **(***n*** = 879)**	**Total** **(***n*** = 1691)**	*p*** value**
n	812	879	1691	
Age at response time, years (median [IQR])	20 [17, 23]	21 [18, 23]	20 [18, 23]	< 0.001
Disease type, n CHD (%)	599 (73.8)	612 (69.6)	1,211 (71.6)	0.067
ADHD, n (%)	49 (6.4)	46 (5.4)	95 (5.9)	0.45
Depression, n (%)	43 (5.6)	72 (8.5)	115 (7.1)	0.035
Anxiety, n (%)	68 (8.9)	165 (19.4)	233 (14.4)	< 0.001
OCD, n (%)	16 (2.1)	44 (5.2)	60 (3.7)	0.0017
Eating Disorder, n (%)	7 (0.9)	22 (2.6)	29 (1.8)	0.020
Migraine, n (%)	68 (8.9)	161 (18.9)	229 (14.2)	< 0.001
Asthma, n (%)	4.5 (5.9)	73 (8.6)	118 (7.3)	0.048

The disease type is congenital heart disease as opposed to acquired cardiovascular disease

*Abbreviations:* *IQR* Interquartile range, using the 25th and 75th quartiles, *CHD* Congenital Heart Disease, *ADHD* Attention-Deficit/Hyperactivity disorder, *OCD* Obsessive–Compulsive Disorder

In the CHD subgroup, the majority (44%) had moderate CHD, 28% had simple CHD, and 25% had severe CHD (3% had unspecified CHD). In this subgroup, 49.5% were males, the median age at the time of answering was 20 years, and the majority (80.3%) reported that they were diagnosed at age 0–5 years. Compared to those with CHD, the acquired heart disease group consisted of relatively fewer males (44.4%) and had a higher median age at the time of answering (22 years). Compared to those with CHD, most of those with acquired heart disease were diagnosed after the age of 5 years (89%).

### Life satisfaction

**Fig. 3:**
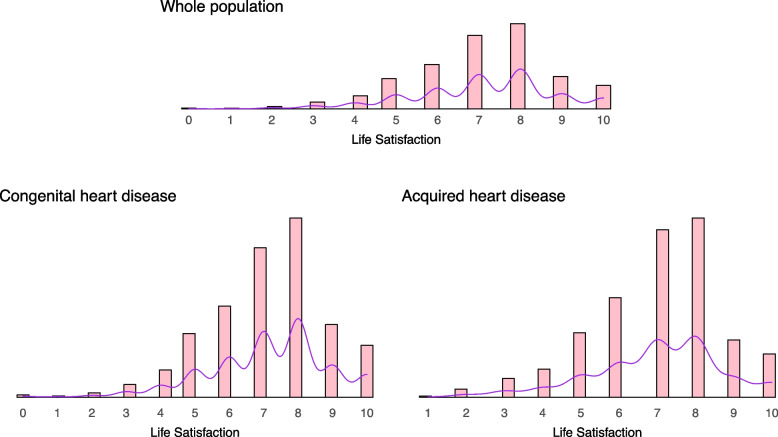
Histogram and density plot of the distribution of life satisfaction, stratified by whole population (upper figure), congenital heart disease subgroup (lower left plot) and acquired heart disease subgroup (lower right plot)

In the study population, 68.8% reported having high life satisfaction (73.6% of males, 64.4% of females, 69.4% of CHD patients, and 67.5% of acquired heart disease patients). The mean life satisfaction was 7.24 [IQR: 6;8] for males, 6.98 [IQR: 6; 8] for females, 7.14 [IQR: 6; 8] for CHD, and 7.01 [6;8] for acquired heart disease. Life satisfaction was normally distributed for the whole population and the subgroups of CHD and acquired heart disease (Fig. 3).

### Association between life satisfaction and challenging factors, self-efficacy, and social support

**Fig. 4:**
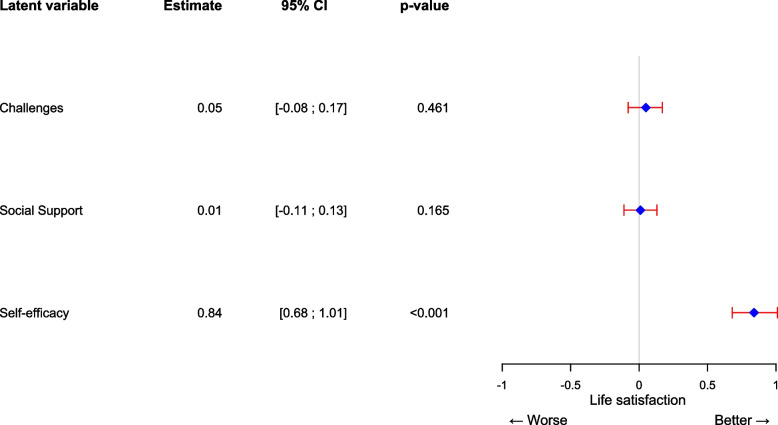
Forest plot depicting the association between challenges, social support, self-efficacy, and life satisfaction. The x-axis depicts the change in the estimate of life satisfaction (diamond) with a 95% confidence interval (red brackets) per standard deviation change in the latent variable on the y-axis. The model shows the association between challenges, social support, self-efficacy, and life satisfaction

In the latent variable model, the correlation estimates between life satisfaction and the latent variables of challenges, self-efficacy, and social support were investigated. In a sensitivity analysis, we stratified by disease type (CHD or acquired heart disease) and found no difference (data not shown), thus, in the final models the whole population was combined. In models with every single latent variable entered separately (social support, self-efficacy, and challenges), we found that high life satisfaction was significantly associated with low challenges (−0.52 [95% CI: −0.56; −0.47], standard error (SE): 0.002, *p*-value < 0.001), high self-efficacy (0.78 [95% CI: 0.73; 0.83], SE: 0.002, *p*-value < 0.001) and high social support (0.38 [95% CI: 0.31; 0.45], SE: 0.035, *p*-value < 0.001). In the mutually adjusted model with all latent variables included, the association between high life satisfaction and high self-efficacy remained significant (0.84 [95% CI: 0.68; 1.01], SE: 0.085, *p*-value < 0.001) (Fig. 4). However, the associations for challenges and social support attenuated toward zero and were no longer statistically significant (challenges: 0.05 [95% CI: −0.08; 0.17], SE: 0.064, *p*-value: 0.461, social support: 0.01 [95% CI: −0.11; 0.13], SE: 0.063, *p*-value: 0.165) (Fig. 4). The model fit indices indicated a good model fit (CFI = 0.93, TLI = 0.909, SRMR = 0.104, and RMSEA = 0.055 (90% CI: 0.049 – 0.060)).

**Fig. 5:**
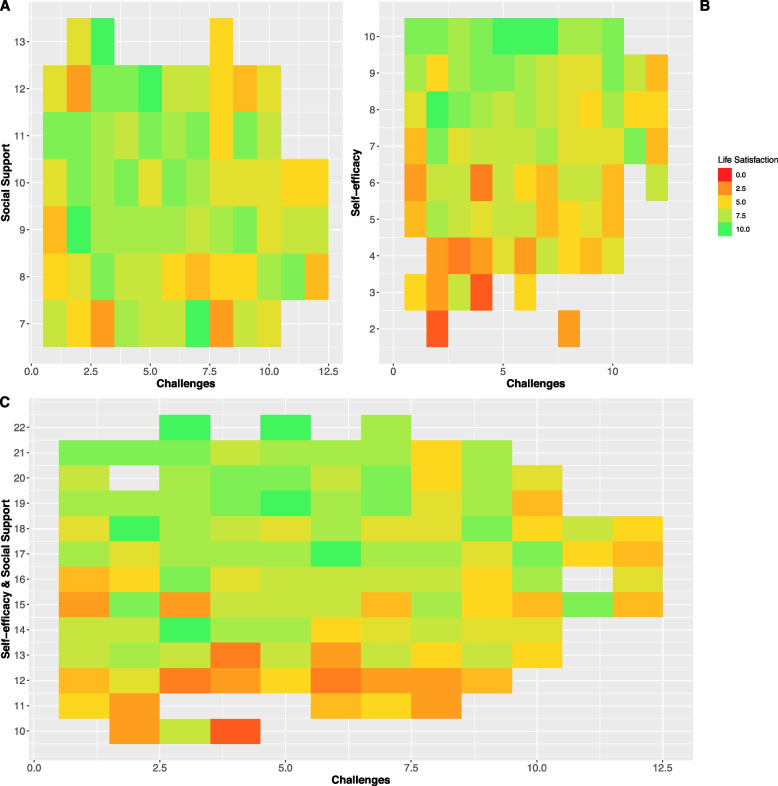
Heatmap plot showing the association between the raw numbers of the exposures used in the model in Fig. 1 and the outcome, life satisfaction. The x-axis in **A**), **B**), and **C**) depicts the combined challenges score, where lower is better. **A**) Y-axis depicts a combined social support score, higher is better. **B** Y-axis depicts a combined self-efficacy score, higher is better. **C**) Combined social support and self-efficacy score, higher is better. The colors depict life satisfaction: green is better, and red is worse (legend)

The data from the survey underlying the latent variable model were described by visualizing the association between the raw numbers of the exposure items and outcome items from the survey. Overall, the same tendencies as the latent variable model were seen (Fig. 5), where low life satisfaction was associated with low self-efficacy and low social support. Overall, these findings support the robustness of the latent variable model.

### Sensitivity analyses

In the sex-stratified analysis, we found similar overall results as our primary model for females and males, with no apparent difference between males and females (correlation between self-efficacy and life satisfaction in males: 0.90 [95% CI: 0.60;1.20], women: 0.84 [95% CI: 0.54; 1.14]). In the age-group stratified analysis, there was no discernable difference between respondents in the young vs. the older age group (correlation between self-efficacy and life satisfaction in the 15–19 years age group: 0.75 [95% CI: 0.20; 0.98], 20–25 years age group: 0.91 [95% CI: 0.68; 1.13]).

In the severity-stratified results, there was a trend of increasing correlation between high self-efficacy and high life satisfaction with increasing CHD severity, however, with overlapping confidence intervals (simple CHD: 0.70 [95% CI: 0.36; 1.03], moderate CHD: 1.02 [95% CI: 0.18; 1.90], severe CHD: 1.25 [95% CI: 0.23; 2.27]), while the associations with both challenges and social support were not statistically significant.

### Nonrespondents

Nonrespondents were less likely to have Danish ethnicity (86% vs 92%), to have an education higher than primary school (62% vs 69%), to be cohabitating with someone (not their parents) (13% vs 16%), and more likely to have parents with limited education (primary school) (11% vs 7%), to be living with a single parent (16% vs 13%), compared with responders.

## Discussion

In this nationwide study of adolescents with CHD or acquired heart disease, high life satisfaction was associated with perceived low levels of cognitive and physical challenges, high self-efficacy, and high social support. In a model considering social support and self-efficacy, challenges were no longer significantly associated with life satisfaction, while high self-efficacy remained independently and statistically significantly associated with high life satisfaction.

### Mechanisms

The positive association between high life satisfaction and high self-efficacy may be mediated by health-promoting behavior that improves life satisfaction [54]. Self-efficacy is a predictor of adherence to physical exercise, treatment, and rehabilitation, as well as of low levels of anxiety and depression [54]. Research on improving self-efficacy in youths often employs varying methods for measuring self-efficacy and varying durations. This lack of standardization complicates comparisons and identification of the best approaches for enhancing self-efficacy. A systematic review by Cataldo et al. demonstrated that interventions involving physical activities significantly improved self-efficacy among youths [55]. These interventions included school-based activities, after-school programs, and camps. Cataldo et al. found that programs featuring supervised physical activities, consistent participation, group settings, and a focus on skill-building or physical capability enhancement led to improvements in self-efficacy. In contrast, another systematic review examined whether youth involvement in decision-making activities, such as organizing activities or serving on councils, found no significant change in self-efficacy post-intervention compared to pre-intervention [56]. Although both reviews were limited by variations in methodologies, the overall findings suggest that group-based physical activities may be the most effective way to improve self-efficacy in youths.

Other studies found that self-efficacy in adolescents can be promoted in different ways, either through social support or through attaining necessary life skills, as well as learning to live with the limitations the chronic disease brings [6, 57–59]. Support from family, place of education, and education programs can foster necessary life skills in these young patients, such as optimism and empowerment [57–59]. Previous studies testing education programs to boost self-management and self-efficacy in adolescents with non-cardiovascular chronic disorders found varying success, although in general they had small sample sizes and a short intervention duration [60–62].

The relationship between social support and life satisfaction may be explained by the resilience theory. Resilience (the ability to recover from adversity) depends on a balance between protective factors and risk factors. One of these protective factors is social support, defined as the presence of others or the resources provided by them, before, during, and following a stressful event [35, 63, 64]. In children with cardiac diseases, high social support is associated with appropriate coping behavior, healthy behavior, feelings of belonging, and a decreased risk of depression [63, 64]. Furthermore, the school or workplace may provide support by providing structure and a sense of normality [65–67]. Adolescents, both with and without CHD, might languish when they feel different, lack structure in their life, and do not feel adequate support [65–67]. This study found that high social support had a significant covariance with high self-efficacy, indicating that social support may play an important role in increasing self-efficacy in young adults, as previously reported [68].

Although challenges and social support were significantly associated with life satisfaction in the univariable model, these associations became non-significant in the full model. Previous studies suggest that higher self-efficacy tends to "moderate" the impact of stressors, particularly those related to social life, in adolescents. In such cases, the negative effects of experienced challenges may have less influence on individuals with high self-efficacy, reducing their need for social support. This reduction may explain why both the associations of challenges and social support became non-significant. Furthermore, we found a significant negative covariance between self-efficacy and challenges, indicating that as one increases, the other diminishes. However, given the observational nature of this study, establishing causality is not possible. Further research is required to explore this hypothesis.

### Comparison with literature

In line with this study, previous studies reported that low physical limitations were associated with both high life satisfaction or high quality of life in children and adolescents with congenital or acquired heart disease [69–72]. However, unlike physical challenges, the association between cognitive challenges and quality of life is marked by inconsistent results, primarily driven by large methodological differences between the studies [15–18]. As such, comparing the results of this study to the literature is difficult, as studies tend to use quality of life and life satisfaction interchangeably [9, 70].

Similar to this study, an association between high self-efficacy and high quality of life has been reported in adolescents with several chronic diseases, including heart disease [18, 73–77]. Likewise, the association between high life satisfaction and high levels of social support from friends, family, school, or workplace in children and adolescents with and without CHD is well described [16, 17, 35, 68, 78].

These findings are not limited to heart diseases. A cross-sectional study looked at the association between self-efficacy and quality of life in adolescence with other types of chronic disorders, including type 1 diabetes, rheumatoid arthritis, cystic fibrosis, urological conditions, or neuromuscular disorders [75]. They found that high self-efficacy was associated with higher quality of life in all domains regardless of the type of chronic disorder. Likewise, we found no difference between youths with CHD or acquired heart disease. Similarly, a studies on healthy youths or youths with different chronic diseases who participated weekly in sports reported higher health-related quality of life and exercise self-efficacy compared to youths who did not participate regularly in sports [55, 79].

With reservations regarding these limitations, high life satisfaction appears to be associated with low levels of physical challenges, high self-efficacy, and high social support.

### Clinical implications

To improve life satisfaction among adolescents with heart disease, our findings underline the importance of promoting self-efficacy and social support, rather than solely focusing on remedying cognitive and physical limitations. Both self-efficacy and social support appear to moderate the role of physical and cognitive challenges in adolescents with heart disease. Interventions that boost both factors could therefore be an important step in improving adolescents’ general satisfaction in life. For instance, it could be camps for adolescents with heart disease focusing on doing certain team-based activities to boost their belief in their own abilities and to socialize. As an example of this is the annual “Football School” held by the Danish Heart Foundation for children with heart disease in collaboration with the Danish Gymnastics and Sports association and some of the biggest football clubs in Denmark [80]. Here children with any or no previous football experience can meet professional football players, get football training, and socialize under the supervision of both professionals and one of their parents.

Furthermore, families, health professionals, places of education, and workplaces should be vigilant in identifying adolescents with seemingly small or non-existent social networks. This may help identify those with low self-efficacy or low life satisfaction, and, if that is the case, the adult may try helping the adolescents in expanding their social network.

The sensitivity analysis of CHD severity found a trend of strong association between self-efficacy and life satisfaction in more severe disease. The more severe chronic disease the patients have, the more symptoms they can experience, both physically and cognitively [50]. Therefore, our findings indicate the importance of improving self-efficacy, especially in youths with moderate or severe CHD. These findings are in line with a study on adult patients with cardiovascular disease, where self-efficacy mediated the relationship between illness severity and health satisfaction [81].

Finally, although the observational nature of this study limits the ability to determine the direction of associations and establishing causality, the findings suggest that future prospective and longitudinal studies should include self-efficacy and specific strategies for improving it when aiming to enhance life satisfaction in adolescents with heart disease. These strategies could involve systematic transition initiatives, educational support, and parental education and involvement. This is particularly important for children with severe congenital heart disease (CHD), where a stronger association between self-efficacy and life satisfaction was observed. Furthermore, longitudinal studies are needed to determine the direction of these associations and to guide future interventions. For instance, researchers could randomize a group of children with heart disease to either an intervention focused on enhancing social support and self-efficacy (e.g., the aforementioned "Football School") or to a control group with no intervention. Annual follow-ups through surveys could then assess whether one group experiences a greater improvement in self-efficacy and life satisfaction over time.

### Strengths and limitations

The main strengths of this study include its large sample size, low risk of selection bias due to random potential participant selection from national registries, and questionnaire development according to recommended practices. This is the first study to provide data on life satisfaction in Danish adolescents with CHD or acquired heart disease, identifying factors that caretakers can identify and monitor.

A limitation of the cross-sectional design is that, although associations between physical and cognitive challenges, self-efficacy, social support, and life satisfaction were found, causality could not be determined. However, the associations themselves were of value, as they may help identify adolescents with heart disease at risk of low life satisfaction. Furthermore, these insights may help guide future interventions aiming to improve life satisfaction in this group by providing potential factors to investigate.

Other limitations pertained to the included sensitivity analyses with wide confidence intervals in sex- and age-stratified analyses, suggesting low power. Moreover, CHD and acquired heart disease patients differed in age at diagnosis, with CHD patients often diagnosed before the age of 5 years, which may have impacted our results [82, 83]. Nevertheless, the distributions of life satisfaction levels were similar between both groups.

Finally, with a 60% response rate, volunteer bias was a potential limitation. Nonrespondents had lower education levels than respondents, possibly affecting generalizability [84, 85]. However, responder and nonrespondent characteristics were overall mostly similar. Furthermore, prior research showed that despite quality of life varying by chronic disorder in adolescents, the association between self-efficacy and quality of life remained consistent across groups [75].

## Conclusions

In this nationwide study, high life satisfaction in adolescents and young adults with congenital or acquired heart disease was associated with low levels of physical and cognitive challenges, high self-efficacy, and high social support. The negative association between life satisfaction and physical and cognitive challenges was attenuated and no longer significant in the presence of high self-efficacy and high social support.

## Supplementary Information

Supplementary Material 1.

## Data Availability

The survey data for this study were accessed through the nationwide registers in Denmark. This data cannot be made publicly available as access must be granted to institutions by the Danish Data Protection Agency and The Danish Health Data Authority. For enquires about the data, please contact the corresponding author.

## Abbreviations

CHD: Congenital Heart Disease
Cis: Confidence intervals
IQR: Interquartile range, using the 25th and 75th quartiles
RMSEA: Root mean square error of approximation
CFI: Comparative fit index
ADHD: Attention-deficit/hyperactivity disorder
OCD: Obsessive-compulsive disorder
