# Supplementary material for: Determinants of life satisfaction in adolescents with congenital or acquired heart disease: a nationwide cross-sectional study
Source: BMC Public Health. 2024 Nov 28;24:3319. doi: 10.1186/s12889-024-20758-5 (PMC11605943; doi:10.1186/s12889-024-20758-5)
Supplement: Supplementary file 1 — Supplementary Material 1. [file 12889_2024_20758_MOESM1_ESM.docx]

**Supplementary material**

Determinants of life satisfaction in adolescents with congenital or acquired heart disease.

El-Chouli et al.

**Table S1:** Diagnosis codes used in this study

|  |  | **ICD-10** |
| --- | --- | --- |
| **Simple CHD** | Ventricular septal defect | Q210 |
|  | Atrial septal defect | Q211 |
|  | Pulmonary stenosis | Q221, Q243 |
|  | Patent ductus arteriosus | Q250 |
| **Moderate CHD** | Congenital mitral insufficiency | Q233 |
|  | Congenital stenosis of aortic valve | Q230, Q244, Q253 |
|  | Congenital insufficiency of aortic valve | Q231 |
|  | Tetralogy of Fallot | Q213, Q218C |
|  | Coarctation of the aorta | Q251 |
|  | Atrioventricular septal defect | Q212, Q218B |
|  | Ebstein's anomaly | Q225 |
|  | Other moderate CHD  (Including more than one simple CHD) | Q214, Q218, Q219, Q222, Q223, Q224, Q226, Q228, Q229, Q238, Q239, Q24 (excluding Q244 and Q246), Q256, Q257, Q26 |
| **Severe CHD** | Congenital malformations of great arteries | Q252, Q254, Q255, Q258, Q259 |
|  | Eisenmenger’s syndrome | Q218A, I278A |
|  | Congenital malformations of cardiac chambers and connections | Q200, Q201, Q202, Q203, Q204, Q205, Q206, Q208, Q209, Q220, Q234, Q242 |
| **Acquired heart disease** | Arrhythmia (supraventricular or ventricular) | I455G, I495, I456, I470, I471G, I471L, I471N, I471P, I471R, I48, I472, I490 |
|  | Heart blocks (atrioventricular or sinus node) | I441, I442, I443, I455B, I455C |
|  | Cardiomyopathies | I42 |
|  | Pulmonary hypertension | I278A |
| **Intellectual disability** |  | Q90, F70-79 |

Abbreviations:
ICD = International Statistical Classification of Diseases and Related Health Problems
CHD = Congenital heart disease

**Table S2**: Item responses in the questionnaire “Adolescence with Heart Disease”

| **Measure** | **Item in the questionnaire** |
| --- | --- |
| **New York Heart Association-Classification** | Which of the following statements below describes best your ability to perform physical tasks   - I: My physical activity is not limited. I can perform ordinary physical activity without experiencing undue shortness of breath, fatigue, or palpitations. - II: My physical activity is slightly limited. I have no discomfort at rest, but regular physical activity (going up the stairs to the 2^nd^ floor, mowing the lawn, vacuuming, or carrying heavy objects) causes me shortness of breath, fatigue, and/or palpitations. - III: I am severely limited in physical activity. I have no discomfort at rest, but light physical activity (walking on a flat road, dressing and undressing, going up the stairs to the 1^st^ floor) causes me pronounced shortness of breath, fatigue, and palpitations. - IV: Discomfort can be present at rest and occurs during any form of physical activity. |
| **Life satisfaction** | “*On this scale, 10 meaning the best life possible for you and 0 meaning the worst life possible for you, where do you currently feel you are?*”  0: Worst possible life  10: Best possible life |
| **Concentration difficulty** | In your daily life, do you have trouble concentrating?   1. Don’t know 2. No, never 3. Yes, but rarely 4. Yes, sometimes 5. Yes, often |
| **Memory difficulty** | In your daily life, do you have trouble remembering what you have to do?   1. Don’t know 2. No, never 3. Yes, but rarely 4. Yes, sometimes 5. Yes, often |
| **Self-confidence** | How much do you agree or disagree with this statement: I am good enough as I am   1. Strongly agree 2. Agree 3. Neither agree nor disagree 4. Disagree 5. Strongly disagree |
| **Belief in own ability** | How often can you accomplish what you set yourself to do?   1. Always 2. Mostly 3. Occasionally 4. Rarely 5. Never |
| **Social support (parents, friends, partner)** | Who do you talk to when something is troubling you or you are upset? (Multiple choices possible)   1. Parents/stepparents    - Yes    - No 2. Siblings    - Yes    - No 3. Friends without heart disease    - Yes    - No 4. Friends with heart disease    - Yes    - No 5. My significant other/partner    - Yes    - No 6. Healthcare personnel    - Yes    - No 7. Personnel at your place of education/school/workplace    - Yes    - No |
